# Signal Transduction Involving the Dmp1 Transcription Factor and its Alteration in Human Cancer

**DOI:** 10.4137/cmo.s548

**Published:** 2008-04-01

**Authors:** Takayuki Sugiyama, Donna P. Frazier, Pankaj Taneja, Robert D. Kendig, Rachel L. Morgan, Lauren A. Matise, Sarah J. Lagedrost, Kazushi Inoue

**Affiliations:** 1 The Department of Pathology, Wake Forest University Health Sciences, Medical Center Boulevard, Winston-Salem, N.C. 27157-0001, U.S.A; 2 The Department of Cancer Biology, Wake Forest University Health Sciences, Medical Center Boulevard, Winston-Salem, N.C. 27157-0001, U.S.A

**Keywords:** Dmp1, Arf, p53, Ras, haplo-insufficiency, lung cancer

## Abstract

*Dmp1* (cyclin D-interacting myb-like protein 1; also called Dmtf1) is a transcription factor that has been isolated in a yeast two-hybrid screen through its binding property to cyclin D2. Dmp1 directly binds to and activates the *Arf* promoter and induces Arf-p53-dependent cell cycle arrest in primary cells. D-type cyclins usually inhibit Dmp1-mediated transcription in a Cdk-independent fashion; however, Dmp1 shows synergistic effects with D-cyclins on the *Arf* promoter. *Ras* or *Myc* oncogene-induced tumor formation is accelerated in both *Dmp1^+/−^* and *Dmp1^−/−^* mice with no significant differences between *Dmp1^+/−^* and *Dmp1^−/−^*. Thus, Dmp1 is haplo-insufficient for tumor suppression. Tumors from *Dmp1^−/−^* or *Dmp1^+/−^* mice often retain wild-type *Arf* and *p53*, suggesting that Dmp1 is a physiological regulator of the Arf-p53 pathway. The *Dmp1* promoter is activated by oncogenic Ras-Raf signaling, while it is repressed by physiological mitogenic stimuli, overexpression of E2F proteins, and genotoxic stimuli mediated by NF-κB. The human *DMP1* gene (h*DMP1*) is located on chromosome 7q21 and is hemizygously deleted in approximately 40% of human lung cancers, especially those that retain normal *INK4a/ARF* and *P53* loci. Thus, h*DMP1* is clearly involved in human carcinogenesis, and tumors with h*DMP1* deletion may constitute a discrete disease entity.

## Introduction

D-type cyclins (D1, D2, and D3) are induced in the context of a delayed early response to growth factor stimulation. Cyclin-dependent kinases 4 and 6 (Cdk4 and Cdk6) are synthesized and assemble with D-type cyclins’ catalytic partners, and both of these processes depend on the presence of mitogens (Giacinti and Giordano, 2006; Sherr, 1996, 2000; Sherr and Robers, 2004). Cyclin D-Cdk holoenzymes have two distinct functions in promoting progression through the G1 phase of the cell division cycle: 1) catalysis of the phosphorylation of the retinoblastoma protein (pRb), and 2) accumulation of cyclin D-Cdk holoenzymes that recruit Cdk inhibitors (such as p27^Kip1^ and p21^Cip1^) into higher order complexes, thereby neutralizing their effects on other Cdks. This process facilitates the activation of cyclin E-Cdk2 later in the G1 phase (Giacinti and Giordano, 2006; Sherr, 1996, 2000; Sherr and Robers, 2004). Among three D-type cyclins, the *Cyclin D1* gene is most commonly involved in human cancers. The human *Cyclin D1* gene is located on chromosome 11q13 and is often amplified (~15%) and overexpressed (~50%) in breast cancers (Arnold and Papanikolau, 2006; Roy and Thompson, 2006). *Cyclin D1* amplification is also found in head and neck, esophageal, and hepatocellular carcinomas and is associated with poor prognosis for patients (for reviews, Donnellan and Chetty, 1997; Sherr, 1996).

The human chromosome 9p21 locus has three different genes that have tumor suppressor functions, namely cyclin-dependent kinase inhibitor 2A (CDKN2A): p16^INK4a^ and p14^ARF^ (*INK4a/ARF*) and CDKN2B: p15^INK4b^, respectively (Sherr, 2004). The former *INK4a/ARF* locus is one of the most frequently disrupted genetic loci in human cancer, the frequency of which is second only to *P53* mutations (Ruas and Peters, 1998). The activity of p53 is positively regulated by p19^Arf^ (p14^ARF^ in humans) in response to oncogenic stress (Lowe and Sherr, 2003; Sherr, 2001, 2006). p19^Arf^ directly binds to Mdm2, thereby stabilizing and activating p53, whereas p16^Ink4a^ binds to cdk4 to inhibit Rb phosphorylation (for reviews, Kim and Sharpless, 2006; Lowe and Sherr, 2003; Sherr, 2001, 2004, 2006). *Arf* is induced by potentially harmful growth-promoting signals stemming from overexpression of various oncoproteins (Lowe and Sherr, 2003; Sherr, 2001). This Arf induction forces early-stage cancer cells to undergo p53-dependent and -independent cell cycle arrest or apoptosis, providing a potent mode of tumor suppression. The *Arf* promoter monitors latent oncogenic signals *in vivo* (Zindy et al. 2003), and accordingly, *Arf*-null mice are highly prone to spontaneous tumor development, especially glioblastomas, carcinomas, and fibrosarcomas (Kamijo et al. 1999). Accumulating evidence has demonstrated the p53-independent functions of Arf (reviewed in Sherr, 2006). The *Arf* promoter is positively regulated by the Dmp1 transcription factor, which will be explained in great detail in this review, and negatively regulated by *Ink4a/Arf* modulators such as Bmi1, Twist, Tbx2/3, and Pokemon (Inoue et al. 2007; Sherr, 2001).

## Isolation of the *Dmp1* Gene

**Figure 1:**
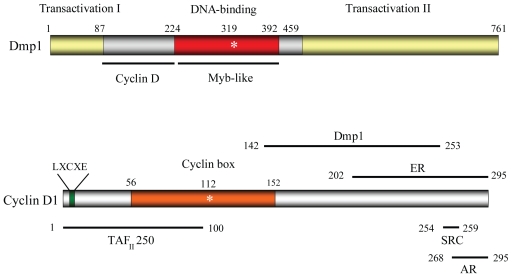
Structure and protein interacting surfaces of Dmp1 and cyclin D1 *Top*. The structure of the Dmp1 transcription factor. Dmp1 consists of 761 amino acids in mice and 760 amino acids in humans, and undergoes extensive posttranslational modification. It has a central DNA-binding domain with three Myb-like repeats that are essential for DNA binding. Mutation of lysine at 319 to glutamic acid abolishes its DNA binding. *Bottom*. Cyclin D1 binds to its catalytic partner Cdk4 through the cyclin box (orange box, amino acid residues 56 to 152). Mutation of lysine at 112 or 114 to glutamic acid abolishes its binding property to Cdks. Cyclin D1 binds to Dmp1 through its carboxyl-terminal half that overlaps the region for estrogen receptor interaction (Inoue and Sherr, 1998; Zwijsen et al. 1997). Cyclin D1 also interacts with TAF_II_250 (Adnane et al. 1999) and androgen receptors (Reutens et al. 2001). **Abbreviations:** ER: estrogen receptor; SRC: steroid receptor coactivator; TAF_II_250: TATA-binding protein-associated factor; AR: androgen receptor.

In 1996, Hirai and Sherr reported the possibility that cyclin D/Cdks might regulate gene expression in an Rb-independent way, suggesting that D-cyclins may involve other genetic programs to facilitate progression of the cell cycle (Hirai and Sherr, 1996). They isolated a novel protein named Dmp1 (cyclin D binding myb-like protein 1; also called Dmtf1, cyclin D binding myb-like transcription factor 1) by using a yeast two-hybrid interactive screen of a murine T-lymphocyte library, with cyclin D2 as bait. The myb gene family consists of three members, named A, B and c-myb which encode nuclear proteins. These proteins function as transcriptional activators or repressors of genes that are involved in cell proliferation, differentiation, apoptosis, and other biological processes. Members of the myb gene family show different temporal and spatial expression patterns suggesting a distinctive function for each of these genes. Loss of the prototype c-myb function in mice results in embryonic lethality due to failure of fetal hepatic hematopoiesis (Oh and Reddy, 1999). Dmp1 binds specifically to the nonameric DNA consensus sequences CCCG(G/T)ATGT to activate transcription (Hirai and Sherr, 1996). Although Dmp1 is related to the myb family proteins for this structure, a subset of these Dmp1 recognition sequences contains a GGA trinucleotide core, a responsive element shared by Ets proteins (Hirai and Sherr, 1996). Inoue and Sherr reported that Dmp1 has a central DNA binding domain that contains three imperfect Myb-like repeats between two acidic transactivation domains. (Fig. 1; Inoue and Sherr, 1998). One experiment with recombinant Dmp1 proteins prepared in Sf9 cells shows that Dmp1 does not need to form homodimers to bind to DNA, although Dmp1 can form homodimers in the absence of DNA (Inoue et al. unpublished data). Dmp1 does not have a clear nuclear localization signal although the protein is localized in the nucleus in transfected as well as in normal cells (Inoue and Sherr, 1998; Mallakin et al. 2006).

The Dmp1 protein migrates at 120–130 kDa although the expected molecular weight is ~ 85 kDa, suggesting the presence of significant post-translational modifications (Hirai and Sherr, 1996; Inoue and Sherr, 1998). D-type cyclins associate with a region of the Dmp1 DNA-binding domain immediately amino-terminal to the tandem Myb-like repeats, to form heteromeric complexes that do not detectably interact with Cdk4 or DNA (Fig. 1; Inoue and Sherr, 1998). Interestingly, the segment of D-type cyclins required for its interaction with Dmp1 was mapped outside the ‘cyclin box’, which contains the residues predicted to contact Cdk4 (Inoue and Sherr, 1998). It was reported that the estrogen receptor binds with cyclin D1 outside the cyclin box (Zwijsen et al. 1997). They showed that cyclin D1 activates estrogen receptor-mediated transcription in the absence of estrogen and enhances transcription in its presence. Interestingly, Reutens et al. reported that the androgen receptor interacts with cyclin D1 through its carboxyl-terminal residues (Reutens et al. 2001). These studies indicated the carboxyl-terminal domain of cyclin D1 plays an important role in physical interactions with DNA-binding proteins, completely independent of cyclin D binding to Cdks (Bernards, 1999; Coqueret, 2002; Inoue et al. 2007). The physiological roles of Cdk-independent functions of cyclin D1 in breast and retinal development has recently been reported (Landis et al. 2006).

Overexpression of the *Dmp1* gene in mouse NIH 3T3 fibroblasts inhibits their entry into S phase (Inoue and Sherr, 1998). Cell cycle arrest depended on the ability of Dmp1 to bind to DNA and transactivate gene expression, and was specifically antagonized by coexpression of D-type cyclins, including cyclin D1 point mutants (D1K112E, K114E) that do not bind to Cdk4 (Inoue and Sherr, 1998; Fig. 1). Studies from other laboratories also showed that overexpression of D-type cyclins inhibited transcriptional activities of other Myb proteins, v-Myb and B-Myb (Ganter et al. 1998; Horstmann, 2000), suggesting a functional link between D-cyclins and Myb-like proteins (Inoue et al. 2007).

## Regulation of the Arf-Mdm2-p53 Pathway by Dmp1

Through extensive search for Dmp1-consensus sequences on naturally occurring promoters, it was found that the murine and human *Arf* promoters and the human *CD13/Aminopeptidase N* have high-affinity Dmp1-binding sequences (Inoue et al. 1998, 1999). Dmp1 directly binds to a unique consensus site 5′-CCCGGATGC-3′ on the murine *Arf* promoter to activate its gene expression (Inoue et al. 1999). Dmp1-mediated *Arf* promoter activation depended on the consensus sequence. When inducible Dmp1:ER virus-infected MEFs (murine embryonic fibroblasts) were stimulated with 4-hydroxytamoxifen (4-HT), both Arf mRNA and protein were upregulated, which induced Arf-, p53-dependent cell cycle arrest within 48 hours (Inoue et al. 1999). E2F1 and Dmp1 showed additive effects on the *Arf* promoter. Since Dmp1 overexpression does not induce apoptosis, it was concluded that p19^Arf^ can be induced in response to anti-proliferative stimuli that do not obligatorily lead to cell death (Inoue et al. 1999).

The mice that lack *Dmp1* have been created by disrupting exons that encode the Myb-like repeats (Inoue et al. 2000). *Dmp1*-null mice are 20%–30% smaller than their wild-type counterparts at birth. Male *Dmp1^−^/^−^* adult mice remained smaller than their wild-type littermates while female *Dmp1*-null adult mice became indistinguishable from their *Dmp1^+^/^+^* or *Dmp1^+^/^−^* littermates. *Dmp1*-null mice have other miscellaneous phenotypes, such as generalized seizures, abnormal seminal vesicle dilatation in males, and poor mammary gland development in females (Inoue et al. 2000).

In cell culture, the growth of *Dmp1*-null MEFs is progressively retarded; however, *p19^Arf^* and p53 levels remain relatively low and the MEFs continued to grow slowly without reaching senescence (Inoue et al. 2000). On the other hand, the rate of p16^Ink4a^ induction in *Dmp1*-null cells remained largely identical to those in *Dmp1^+^/^+^* and *Dmp1^+^/^−^* cells. Intriguingly, the levels of Dmp1 increased from passage 2 to passage 3 in both *Dmp1^+^/^+^* and *Dmp1^+^/^−^* cells, and the accumulation of *Dmp1* preceded that of p19^Arf^ (Inoue et al. 2000). This data suggested that stress signaling caused by non-physiological cell culture conditions induces *Dmp1*, which in turn activates p19^Arf^. When wild-type MEFs were continuously cultured over the period of replicative senescence, immortalized cell lines that had either a mutant *p53* (~80%) or deleted *Arf* locus (~20%) were obtained (Inoue et al. 2000). In long term culture, *Dmp1^−^/^−^* cells readily gave rise to established cell lines that retained wild-type *Arf* and functional p53 without overexpression of Mdm2, suggesting that the activity of the Arf-Mdm2-p53 pathway is significantly attenuated in *Dmp1^−^/^−^* cells (Inoue et al. 2000). Hence *Dmp1*-deficient MEFs were morphologically transformed by Ha*-*Ras^V12^ alone (Inoue et al. 2000).

## Tumor Formation in *Dmp1*-Deficient Mice

**Figure 2:**
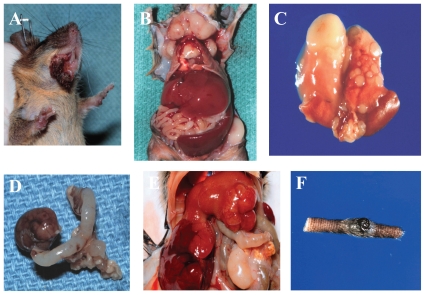
Tumors found in *Dmp1*-deficient mice **A:** mandibular carcinosarcoma (*Dmp1^−/−^*, untreated, 76 weeks); **B:** T-cell leukemia/lymphoma (*Dmp1^+/−^*, untreated, 48 weeks); **C:** lung adenocarcinoma (*Dmp1^−/−^*, untreated, 86 weeks); **D:** ovarian granulose cell tumor (*Dmp1^−/−^*, DMBA-treated, 26 weeks); **E:** hepatocellular carcinoma (*Dmp1^+/−^*, irradiated, 61 weeks); and **F:** malignant melanoma (*Dmp1^−/−^*, DMBA-treated, 39 weeks). These tumors were not observed in the *Dmp1^+/+^* littermate controls of the same age (Inoue et al. 2000, 2001).

The *Dmp1*-null mice developed tumors in their second year of life with a mean latency of 83 weeks (Inoue et al. 2001). These *Dmp1*-null mice spontaneously developed pulmonary adenomas/adeno-carcinomas (42%), vascular tumors (hemangiomas and hemangiosarcomas) (24%), liver tumors (hepatocellular adenomas/adenocarcinomas) (18%) and B-cell lymphomas (15%) (Inoue et al. 2001; Fig. 2). The time of tumor onset and the spectra of tumors observed in *Dmp1*-null mice bore no obvious relationship to those in *Arf*-null or *p53*-null mice, which exhibit a different spectrum (Kamijo et al. 1999; Donehower et al. 1992). Treatment of neonatal *Dmp1*-deficient mice with dimethylbenzanthracene (DMBA) or ionizing radiation helped to develop multiple tumors, including lung, skin, and liver carcinomas, T-cell leukemia/lymphomas, and ovarian tumors (Inoue et al. 2000, 2001; Fig. 2). The control *Dmp1^+^/^+^* mice that had received the same treatment were not found to have these tumors. These results suggest that Dmp1-inactivation clearly contributed to the change the tumor spectra. In humans, epithelial tumors such as adenocarcinomas are usual and carcinomas are commonly found after 40 years of age. Since *Dmp1*-null mice developed epithelial tumors in their second year of life, the *Dmp1*-knockout mice may be useful carcinogenesis models for adult humans.

## Haploid Insufficiency of Dmp1 in Tumor Suppression

When crossed onto a *Dmp1^+^/^−^* or *Dmp1^−^/^−^* background, lymphomas induced by the E*μ*-*Myc* trans-gene were greatly accelerated (mean latency, 12 weeks) with no differences between cohorts lacking one or two *Dmp1* alleles (Inoue et al. 2001). The latency in the *Dmp1^+^/^−^* or *Dmp1^−^/^−^* strains is similar to that of *Arf* *^+^/^−^*, E*μ-Myc* transgenic mice (Eischen et al. 1999; Inoue et al. 2001). These results consistently suggest that Dmp1 loss lowers p19^Arf^ expression (Inoue et al. 1999, 2000). On the other hand, tumors from *Dmp1*-heterozygotes retained and expressed the wild-type *Dmp1* allele and expressed detectable Dmp1 protein (Inoue et al. 2001). In five of these tumors, nucleotide sequencing by using reverse transcription-polymerase chain reaction products showed no mutations in the DNA-binding domain in Dmp1. These results clearly confirm that Dmp1 is haplo-insufficient for tumor suppression (Inoue et al. 2001, 2007; for reviews, Brooksbank, 2001; Quon and Berns, 2001). Importantly, the combined frequencies of *p53* mutation and *Arf* deletion in the *Dmp1^−^/^−^* and *Dmp1^+^/^−^* cohorts were ~10%, versus ~50% in *Dmp1^+^/^+^* littermates, suggesting that Dmp1 is a physiological regulator of the Arf-p53 pathway in living animals.

## Activation of *Dmp1* Transcription by Oncogenic Ras-Raf Signaling

Ras-mediated signaling pathways play critical roles in the mitogen-dependent induction of cyclin D1 and its assembly with Cdk4 (Cheng et al. 1998). Overexpression of activated Ras stimulates DNA synthesis independent of growth factor stimulation. Conversely, continuous overexpression of oncogenic Ras and its various effectors can lead to irreversible cell cycle arrest by upregulating the levels of p16^Ink4a^, p19^Arf^, and p53 (Lin et al. 1998; Palmero et al. 1998; Serrano et al. 1997; for review McMahon and Woods, 2001). It has been speculated that transcriptional control plays an important role in Dmp1 regulation because the Dmp1 protein has a relatively long half-life (~12 hours) (Mallakin et al. 2006). In cultured primary cells, the *Dmp1* promoter was efficiently activated by oncogenic Ha-Ras^V12^ but not by overexpressed c-Myc or E2F-1 (Sreeramaneni et al. 2005). Double mutant Ras^V12S35^ activated the *Dmp1* promoter, and MEK/ ERK inhibitor U0126 completely blocked the *Dmp1* promoter activation, indicating that the *Dmp1* promoter activation by Ras^V12^ depended on Raf-MEK-ERK signaling (Sreeramaneni et al. 2005). Consistently, *Dmp1*-null cells were resistant to Raf-mediated premature senescence, which showed significantly decreased induction of p19^Arf^ and p21^Cip1^ by oncogenic Raf (Sreeramaneni et al. 2005). These results revealed that Dmp1 is a critical target for oncogenic Raf-induced premature senescence. Importantly, a Ras^V12^-responsive element was located onto the 50-base-pair leader sequence of the murine *Dmp1* promoter, where endogenous Fos and Jun family proteins bind. The *Dmp1* promoter activation by Ras^V12^ was significantly attenuated in c-*Jun* as well as in *JunB* knockdown cells, suggesting Jun proteins have a critical role in *Dmp1* promoter activation (Sreeramaneni et al. 2005). It is generally believed that c-Jun is phosphorylated by JNK/SAPK, the activity of which is regulated by the MEKK1-MEK4/7 pathway. This MEKK1-MEK4/7-JNK/SAPK signaling is different from the classical Raf-MEK1/2-ERK1/2 pathway (Johnson and Lapadat, 2002; Kallunki et al. 1996; Shaulian and Karin, 2002). However, it has also been reported that oncogenic Ras activates MEKK1 (Marshall, 1995). Thus, c-Jun phosphorylation is regulated by Ras signaling. On the other hand, the *c-Jun* promoter is regulated by the Ras-Raf-MEK1/2-ERK1/2-MSK-ATF1 pathway (Gupta and Prywes, 2002). For JunB, it has been reported that activated p44^ERK-1^ enhances Ets-mediated transactivation of the *JunB* promoter in response to Ras signaling, but JNKs do not phosphorylate JunB (Coffer et al. 1994; Kallunki et al. 1996). Thus, oncogenic Ras regulates the *Dmp1* promoter both by transcriptional activation of the c-*Jun/JunB* promoters and by phosphorylation of the c-Jun protein.

**Figure 3:**
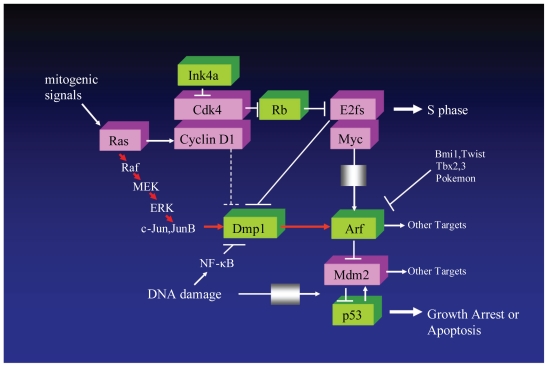
Signaling pathways involving Dmp1 Arf is induced by potentially oncogenic signals stemming from overexpression of oncogenes such as c-Myc, E2F-1, and activated Ras. This induction quenches inappropriate mitogenic signaling by diverting incipient cancer cells to undergo p53-dependent and -independent growth arrest or cell death. *Arf* expression is repressed by a number of nuclear proteins, such as Bmi1, Twist, Tbx2/3, and Pokemon. Dmp1 is unique in that it directly binds and activates the *Arf* promoter and induces cell cycle arrest in an *Arf*-dependent fashion. Both *Dmp1*-null and heterozygous mice show hypersensitivity to develop tumors in response to carcinogen DMBA and *γ*-irradiation. This phenotype could be explained by the inactivation of the Arf-Mdm2-p53 pathway in the absence of the functional Dmp1 protein, although it is possible that Dmp1 has targets other than *Arf.* D-type cyclins inhibit Dmp1’s transcriptional activity in a Cdk-independent fashion when E2Fs do not bind to the same promoter; however, D-cyclins cooperate with Dmp1 to activate the *Arf* promoter. The *Dmp1* promoter is efficiently activated by the oncogenic Ras-Raf-MEK-ERK-Jun pathway but is repressed by overexpressed c-Myc, E2Fs, and by physiological mitogenic signaling. The induction of Arf by oncogenic Ras is largely dependent on Dmp1. We recently reported that the Dmp1-Arf pathway was inhibited by NF-κB proteins in response to genotoxic stress signaling (Taneja et al. 2007).

A Ras^V12^-responsive element was mapped to the unique Dmp1/Ets site on the *Arf* promoter, where endogenous Dmp1 proteins bind after oncogenic Raf activation (Sreeramaneni et al. 2005). Although oncogenic Ras indirectly activates E2F transcription factors, E2Fs do not play an important role in *Arf* induction by Ras^V12^ (Palmero et al. 2002; Rowland et al. 2002). Therefore, the *Arf* promoter activation induced by Ras/Raf signaling is mediated by Dmp1, and this is why *Dmp1*-null primary cells are highly susceptible to Ras-induced transformation (Inoue et al. 2000). We proposed that the novel Jun-Dmp1 pathways directly links oncogenic Ras-Raf signaling and p19^Arf^, independent of the classical cyclin D1/Cdk4-Rb-E2F pathway (Fig. 3; Sreeramaneni et al. 2005).

Overexpressed D-type cyclins usually antagonize Dmp1’s transcriptional activity in a Cdk-independent fashion when tested with artificial promoter-reporter plasmids (containing con-catamerized Dmp1 consensus binding sequences), or with some natural occurring promoters (such as those derived from the *CD13/Aminopeptidase N* gene) (Inoue et al. 1998). However, the results were reversed for the *Arf* promoter where D-type cyclins cooperated to enhance the activity of Dmp1 in a Cdk4-dependent manner. The *Arf* promoter has both Dmp1- and E2F-binding sites, enabling Ras^V12^-induced cyclin D1 to assemble with Cdk4, promote to release E2Fs from pocket proteins, and then collaborate with Dmp1 in activating *Arf* gene expression (Inoue et al. 1999; Sreeramaneni et al. 2005). On the other hand, the *CD13/Aminopeptidase N* promoter, which lacks E2F-consensus sequences, can be suppressed by D-type cyclins, which can interfere with Dmp1 binding to DNA when overexpressed. Interestingly, the Dmp1/Ets-consensus sequences found within these two promoters are completely identical (CCCGGATGC) (Inoue et al. 1998, 1999). Thus, the sequences flanking the Dmp1-binding site determine the responsiveness of the promoter to D-type cyclins.

## Negative Regulation of the *Dmp1* Promoter: Repression by E2Fs and NF-κB

In wild-type MEFs, the *Arf* promoter is occupied by E2F3 and not other E2F family members. In quiescent cells, this role is largely fulfilled by E2F3b, an E2F3 isoform whose function was previously undetermined (Aslanian et al. 2004; for E2F review, Taneja et al. 2007; Trimarchi and Lees, 2002). On the other hand, endogenous activating E2Fs, E2F1 and E2F3a are recruited to the *Arf* promoter in response to hyperproliferative oncogenic signaling, indicating that distinct subsets of E2F proteins contribute to physiological repression and oncogenic activation of *Arf* (Aslanian et al. 2004). The *Dmp1* promoter was efficiently repressed by overexpression of E2F1, E2F2, E2F3a, E2F3b, and E2F4 as well as physiological mitogens (Mallakin et al. 2006). Chromatin immunoprecipitation demonstrated the binding of endogenous E2Fs on the *Dmp1* promoter when synchronized cells entered the S phase of the cell cycle (Mallakin et al. 2006). E2F-DB is a mutant of E2F1 that lacks a transactivation domain. Disruption of transcriptional repressor complexes with E2F-DB causes a general increase of E2F target genes, but the cells become immortalized and are resistant to senescence by p19^Arf^, p53, and Ras^V12^ (Rowland et al. 2002). The *Dmp1* mRNA was not downregulated in E2F-DB(+) cells in response to serum, suggesting that the *Dmp1* promoter repression by serum was E2F-dependent (Mallakin et al. 2006). Thus, E2F1 has differential effects on the *Dmp1* promoter (repression) and the *Arf* promoter (activation) when overexpressed in rodent fibroblasts (Inoue et al. 1999; Mallakin et al. 2006). The h*DMP1* promoter also has a typical E2F site and is efficiently repressed by E2Fs (Mallakin et al. 2006). Although the mechanisms by which non-physiological E2F expression have differential effects on the *Dmp1* and *Arf* promoters are not clear, the simplest explanation is that the differences of the flanking DNA sequences around the E2F sites determine the responsiveness of each promoter to E2Fs since distinct cofactors will bind to each promoter. Of note, the *Dmp1* promoter is not the only one that is repressed by ‘activating’ E2Fs; repression of the human *telomerase* promoter, tumor suppressor *ARHI* promoter, and *PNRC2* (Proline-rich Nuclear Receptor Coactivator 2) promoter by E2F1 have been reported (Crowe et al. 2001; Lu et al. 2006; Zhou et al. 2005).

The Dmp1 is expressed in the testis, thymus, spleen, lung brain, and intestines (Inoue et al. 2000; Mallakin et al. 2006). In order to identify Dmp1-expressing cells *in vivo*, immunohistochemical stainings were performed to disclose the Dmp1 expression pattern in normal murine tissues compared with the proliferation marker Ki67 (Mallakin et al. 2006). Ki67 antigen is the prototypic cell cycle-related nuclear protein, expressed by proliferating cells in all phases of the active cell cycle (G1, S, G2 and M phase) (Brown and Gatter, 2002). It is absent in resting (G0) cells. Thus, Ki67 antibodies are useful in establishing the cell growing fraction in neoplasms. The correlation between high Ki67 index and histologically high grade tumors is strong. High Ki67 index is associated with poor prognosis of a variety of human cancers (Brown and Gatter, 2002; Diest et al. 2004). In the thymus, nuclei of mature T lymphocytes in the medulla were strongly positive for Dmp1, whereas Ki67 was detected only in the cortex. In the intestines, Dmp1 was detected in the nuclei of superficial layers of the villi, whereas Ki67-positive cells were confined to the bottom of the crypt. Double staining for Dmp1 and Ki67 revealed that these two proteins were expressed in a mutually exclusive fashion in nearly all of the tissues examined (Mallakin et al. 2006). The prototype of Dmp1, the c-Myb protein, is abundantly expressed in the thymic cortex (Oh and Reddy, 1999). Thus, c-Myb and Dmp1 may play complementary roles in regulating the gene expression involved in cell growth and differentiation.

The *Dmp1* and *Arf* promoters receive non-oncogenic signals as well. Both genotoxic and oncogenic stress activates the nuclear factor-kappa B (NF-κB) and p53 proteins; however, p53 activity is antagonized by NF-κB signaling (Perkins, 2004). Among NF-κB proteins, the p65, p50, and p52 subunits are ubiquitously expressed whereas the RelB and c-Rel subunits are relatively specific to lymphoid/hematopoietic tissues (Carrasco et al. 1993; Hayden and Ghosh, 2004). The *Dmp1* promoter was repressed by treatment of cells with anthracyclins and UV-C; non-classical NF-κB activators (Taneja et al. 2007; Fig. 3). Following anthracyclin/UV-C treatment, p65 and other subsets of NF-κB proteins were bound to the *Dmp1* promoter (Taneja et al. 2007). Repression of *Dmp1* transcription by anthracyclins depended on the unique NF-κB site on the promoter. Among NF-κB proteins, p65 played the major role in *Dmp1* repression since downregulation of p65 by shRNA significantly attenuated the response of the promoter by anthracyclins/UV-C. The amount of Dmp1 bound to the *Arf* promoter decreased significantly upon anthracyclin treatment; this treatment, in turn, downregulated the p19^Arf^. Repression of the *Arf* promoter by p65 or anthracyclins depended on Dmp1, which was almost absent in *Dmp1^−^/^−^* cells (Taneja et al. 2007). Compared to wild-type cells, both *Dmp1^−^*/*^−^* and *Arf* *^−^*/*^−^* cells showed resistance to anthracyclin-induced cell death. Non-immortalized *p65*-knockdown cells were much more sensitive to anthracyclins than wild-type cells, indicating the role of p65 in protecting cells from apoptosis (Taneja et al. 2007). Thus, the Dmp1-Arf pathway is repressed by NF-κB in response to genotoxic stress, which implicates a novel mechanism of p53 inactivation by NF-κB (Fig. 3).

What is the significance, then, of inhibition of the Dmp1-Arf signaling by genotoxic stress? Doxorubicin treatment of cells stimulates nuclear accumulation and phosphorylation of p53, a process that is mediated by ATM (Kurz et al. 2004). Taneja et al. showed that Dmp1-Arf and p53 were differentially regulated by anthracyclins for at least 4 hrs after drug treatment; however, both p53 and p21^Cip1^ decreased when Dmp1 and p19^Arf^ were downregulated (Taneja et al. 2007). Thus, attenuation of the Dmp1-Arf pathway by anthracyclins appears to mediate protection of normal cells from the extensive cell death induced by genotoxic drugs. This mechanism will be especially important with respect to the side effects of chemotherapeutic agents in normal tissues. The major mechanisms of action of anthracyclins are considered to be stabilization of topoisomerase IIα cleavage complexes and generation of reactive oxygen intermediates (DeVita et al. 2005). The former causes protein-linked double- and single-stranded DNA breaks, which lead to cyto-toxic DNA damage and cell death. Thus, cancer cells are generally much more sensitive to anthracyclins than normal tissues even when they have *ARF* deletions or *P53* mutations, simply because they divide more frequently than normal cells.

## The Human *DMP1* (h*DMP1*) Gene

Bodner et al. sequenced three independent EST (expressed sequence tag) clones for the h*DMP1* gene and reported that the human DMP1 protein consists of 760 amino acids. The DMP1 protein has very high structural homology with its murine counterpart (96% similarity at amino acid levels), and the sequence of the three myb-like repeats is completely identical. The h*DMP1* gene is located on human chromosome 7q21, a locus often deleted in some human carcinomas and hematopoietic malignancies (Bieche et al. 1992; Bodner et al. 1999; Kerr et al. 1996; Trovato et al. 2004). Interestingly, the FISH analysis of leukemic samples with abnormalities on chromosome 7 showed that one allele of the h*DMP1* gene was deleted in 9 of 9 cases, suggesting the involvement of the DMP1 locus in 7q- leukemias (Bodner et al. 1999).

Tschan et al. reported that the locus of h*DMP1* encodes at least three splicing variants (h*DMP1α*, *β* and *γ*) (Tschan et al. 2003). The h*DMP1β* and h*DMP1γ* isoforms have been cloned by RT-PCR, using the cDNA library of KG-1 cells. The open reading frames of h*DMP1β* and h*DMP1γ* encode identical initial amino acid (aa) sequences to h*DMP1*α up to the splice site at aa 237. However, after aa 237, *h*DMP1β and *h*DMP1γ show novel sequences of 35 and 48 aa, respectively, followed by a premature stop codon occurring in the alternatively spliced intronic sequence. The *h*DMP1β and γ isoforms still contain the acidic N-terminal transactivation, the cyclin D binding, and a part of the Myb-homology domains but no C-terminal transactivation domain. The predicted length of proteins encoded by h*DMP1α*, -*β* and -*γ* are 760, 272, and 285 aa, respectively. The β- and γ-splicing variants do not bind to DNA, but they can inhibit transactivation of the *CD13/Aminopeptidase N* promoter by *h*DMP1α (Tschan et al. 2003). The full-length *h*DMP1α corresponds to murine Dmp1, which directly binds to the *Arf* promoter and positively regulates the p19^Arf^-p53 pathway (Inoue et al. 1999). Therefore, it has been speculated that *h*DMP1α engages in tumor-suppressor activity. The h*DMP1β* and *γ* genes are specifically expressed in immature hematopoietic cells. Interestingly, U937 cells that constitutively express h*DMP1β* isoform showed reduced cell surface expression of CD13/Aminopeptidase N and continued to proliferate even after phorbol 12-myristate 13-acetate treatment (Tschan et al. 2003). Therefore, it was suggested that splicing abnormalities of h*DMP1* that result in the overexpression of h*DMP1βγ* isoforms may contribute to human leukemogenesis (Tschan et al. 2003).

## Dmp1 and Lung Cancer: From Mouse Models to Human Disease

As mentioned earlier, the *Dmp1* promoter receives oncogenic signaling from mutant Ras. The *K-ras^LA/+^* (*K-ras^LA1/+^*, *K-ras^LA2/+^*) mouse model is one of the most sophisticated models that mimics human non-small cell lung cancer (NSCLC) (Johnson et al. 2001; for comprehensive review of mouse models of lung cancer, see Meuwissen and Berns, 2005). In this model, the *K-ras* gene is controlled by its own promoter and is activated during spontaneous recombination events in the whole animal (Johnson et al. 2001). On the other hand, *Dmp1*-knockout mice are prone to tumor development, especially lung adenocarcinomas (Inoue et al. 2000, 2001). Based on this information, we crossed *Dmp1*-deficient mice with *K-ras^LA^* mice and reported that *K-ras^LA^*-induced lung carcino-genesis was significantly accelerated in both *Dmp1^+^/^−^* and *Dmp1^−^/^−^* mice, with little difference between the two cohorts (Mallakin et al. 2007). The lung tumor cells from *Dmp1^+^/^−^*; *K-ras^LA^* mice expressed *Dmp1* mRNA 2–4 times higher than in lungs from *Dmp1^+^/^−^* mice in most cases, suggesting endogenous *Dmp1* promoter activation by oncogenic *K-Ras* (Mallakin et al. 2007). Our report suggested the haploid-insufficiency of Dmp1 in lung cancer suppression.

*K-ras^LA^* lung tumors are different from Eμ-*Myc* lymphomas in that neither bi-allelic *Arf* deletion or Mdm2 overexpression were found in any tumors, regardless of the genotype of *Dmp1* (Eischen et al. 1999; Inoue et al. 2001; Mallakin et al. 2007). None of the *Ink4a/Arf* modulators, such as Bmi1, Twist, Tbx2/3, and Pokemon were overexpressed in *K-ras^LA^* lung tumors, ruling out the possibility of the involvement of these *Ink4a/ Arf* modulators for *K-ras*-induced tumor formation. *p53* mutation was less frequent in lung tumors from *Dmp1^+^/^−^, Dmp1^−^/^−^; K-ras^LA^* mice, thus *Dmp1* deletions and *p53* mutations might have similar effects. In fact, we have found that tumors that showed deletion of *Dmp1* tended to show the phenotype of adenocarcinomas (5/7, 71%) (Mallakin et al. 2007). All the lung tumors that showed mutation of *p53* were adenocarcinomas (4/4, 100%). On the other hand, lung tumors that did not show *Dmp1* or *p53* alterations were mostly adenomas, and there was only one case of adenocarcinoma in this group (1/5, 20%). Thus, deletions of *Dmp1* or mutations of *p53* are frequently associated with malignant phenotypes of *K-ras^LA^* lung tumors.

**Figure 4:**
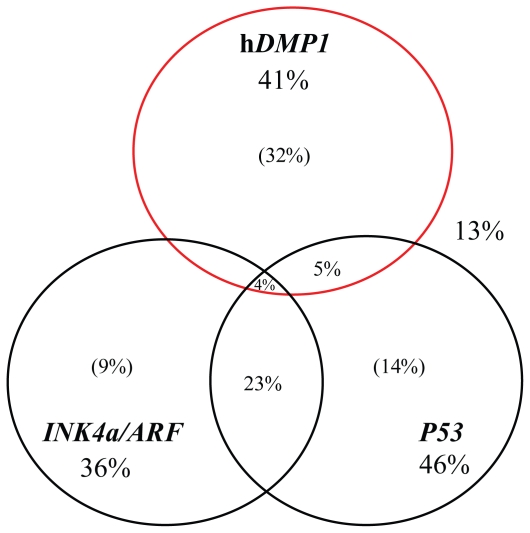
Deletion of h*DMP1* is a new category of human lung cancer Fifty-one cases of human non-small cell carcinoma (NSCLC) were studied for loss of heterozygosity (LOH) with 6 sets of PCR primers (2 sets for the h*DMP1* locus, 2 sets for the *INK4a/ARF* locus, and 2 sets for the *P53* locus) (Mallakin et al. 2007). The numbers show the percentage of lung cancer samples that showed LOH for each tumor suppressor locus with our relaxed criteria, i.e. the LOH values showed >2.0 or <0.5 with one of the two sets of primers. The numbers in parenthesis show the percentages of LOH cases that do not overlap LOH of other loci. Eighty-seven percent of NSCLC showed LOH with at least one of these sets of primers. LOH of h*DMP1* occurred in a mutually exclusive fashion with LOH of *INK4a/ARF* or that of *P53* in most cases. On the other hand, a significant percentage of samples showed LOH for both *INK4a/ARF* and *P53*.

In human lung cancers, *p14^ARF^* is inactivated in 65% of small-cell lung cancer (SCLC), while the gene is deleted in ~20% of NSCLC. Promoter hypermethylation of *ARF* has been reported in ~10% of NSCLC, but is less frequent than that of *p16^INK4a^* (~40%) on the same locus (Meuwissen and Berns, 2005). We recently analyzed loss of heterozygosity (LOH) of h*DMP1*, *INK4a/ARF*, and *P53* in more than 50 cases of human NSCLC samples (Mallakin et al. 2007). LOH of h*DMP1* was found in ~35% (41% if we use relaxed criteria) of NSCLC (Fig. 4). Interestingly, the LOH of the h*DMP1* locus and that of the *INK4a/ARF* or *P53* loci occurred in a mutually exclusive fashion, consistent with the hypothesis that hemizygous deletion of h*DMP1* may inactivate the ARF-P53 pathway (Mallakin et al. 2007; Fig. 4). The region that was deleted in human lung cancer was limited to the h*DMP1* locus in ~80% of the cases, indicating that lung cancer cells specifically target the h*DMP1* gene (Mallakin et al. 2007). Point mutations, promoter hypermethylations, and splicing alterations that result in h*DMP1β* overexpression were not common in human NSCLC. The *h*DMP1 protein was very low or barely detectable in the nuclei of NSCLC cells that showed LOH of h*DMP1* (Mallakin et al. 2007). Interestingly, expression and activation of Dmp1:ER in the *ARF*^+^ *P53* wild-type lung cancer cell line strongly inhibited the growth of the cells, while other lung cancer cells with deletion of *ARF* or *P53* were relatively resistant (Mallakin et al. 2007). Thus, it is highly possible that the h*DMP1* gene is inactivated in a significant percentage of other types of human cancers, especially those that retain wild-type *ARF* and *P53*. Hence, ‘reactivation’ of the h*DMP1* gene in cancer cells might be a feasible approach for novel cancer therapy since tumor cells often have one intact h*DMP1* allele.

## Conclusion and Future Directions

Dmp1 is a Myb-like transcription factor that is haplo-insufficient for tumor suppression and is a physiological regulator of the Arf-p53 pathway. The *Dmp1* promoter is activated by oncogenic Ras-Raf signaling, but is inhibited by physiological mitogen, aberrant E2F expression, and geno-toxic stimuli mediated by NF-κB. It will be helpful to conduct double staining of Dmp1 vs. c-Jun/ JunB, E2Fs, and NF-κB in tissues to understand their relationships during cell growth and differentiation. In contrast to the accumulation of information on the *Dmp1* promoter, very little is known about the Dmp1 protein. Future studies will clarify the mechanism of post-translational modifications and identification of novel binding and transcriptional targets of Dmp1. Our recent study shows that the h*DMP1* gene is deleted in a significant percentage of human lung cancers, indicating its primary involvement in human carcinogenesis. Judging from the tumor spectra of *Dmp1*-knockout mice, it should be involved in a variety of malignancies. Thus, it will be crucial to study inactivation/aberrant expression of h*DMP1* in a broad spectrum of human cancers, and correlate the results with patients’ prognoses to apply the results of basic studies to clinical levels.
